# CTLA-4—two pathways to anti-tumour immunity?

**DOI:** 10.1093/immadv/ltaf008

**Published:** 2025-03-07

**Authors:** Frank J Ward, Paul T Kennedy, Farah Al-Fatyan, Lekh N Dahal, Rasha Abu-Eid

**Affiliations:** Medical Sciences and Nutrition, Institute of Medical Sciences, School of Medicine, University of Aberdeen, Aberdeen, United Kingdom; Department of Pharmacology and Therapeutics, University of Liverpool, Liverpool, United Kingdom; Medical Sciences and Nutrition, Institute of Medical Sciences, School of Medicine, University of Aberdeen, Aberdeen, United Kingdom; Department of Pharmacology and Therapeutics, University of Liverpool, Liverpool, United Kingdom; Medical Sciences and Nutrition, Institute of Dentistry, School of Medicine, Sciences & Nutrition, University of Aberdeen, Aberdeen, United Kingdom; School of Dentistry, College of Medicine and Health, The University of Birmingham, Birmingham, United Kingdom

**Keywords:** immune checkpoint inhibitor, CTLA-4, regulatory T cells (Treg), soluble CTLA-4 (sCTLA-4)

## Abstract

Immune checkpoint inhibitor (ICI) therapies have revolutionized cancer therapy and improved patient outcomes in a range of cancers. ICIs enhance anti-tumour immunity by targeting the inhibitory checkpoint receptors CTLA-4, PD-1, PD-L1, and LAG-3. Despite their success, efficacy, and tolerance vary between patients, raising new challenges to improve these therapies. These could be addressed by the identification of robust biomarkers to predict patient outcome and a more complete understanding of how ICIs affect and are affected by the tumour microenvironment (TME). Despite being the first ICIs to be introduced, anti-CTLA-4 antibodies have underperformed compared with antibodies that target the PD-1/PDL-1 axis. This is due to the complexity regarding their precise mechanism of action, with two possible routes to efficacy identified. The first is a direct enhancement of effector T-cell responses through simple blockade of CTLA-4—‘releasing the brakes’, while the second requires prior elimination of regulatory T cells (T_REG_) to allow emergence of T-cell-mediated destruction of tumour cells. We examine evidence indicating both mechanisms exist but offer different antagonistic characteristics. Further, we investigate the potential of the soluble isoform of CTLA-4, sCTLA-4, as a confounding factor for current therapies, but also as a therapeutic for delivering antigen-specific anti-tumour immunity.

## Graphical Abstract

**Graphical Abstract:**
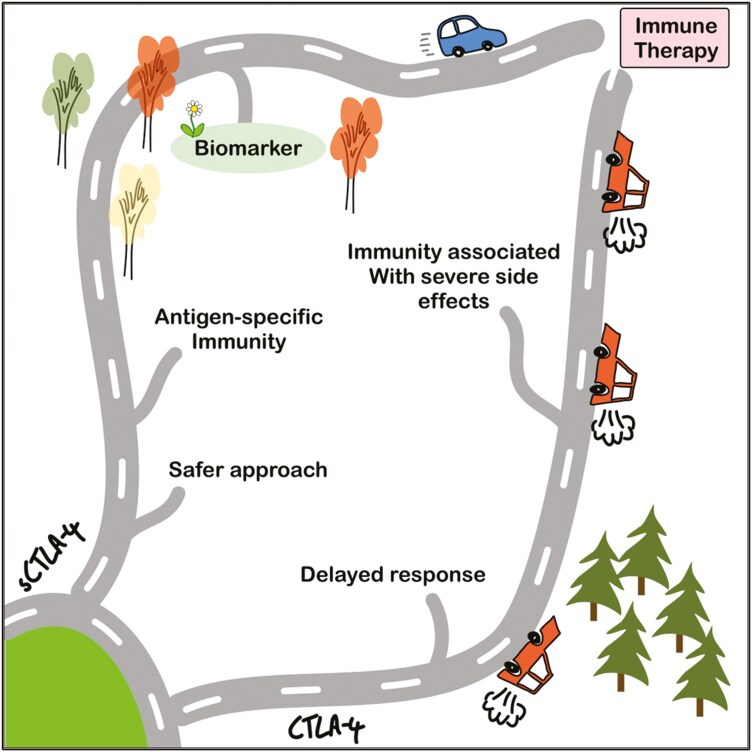

## Introduction

Checkpoint inhibitor immunotherapies (ICIs) that modulate T-cell function offer new and exciting prospects for the treatment of cancer [1, 2]. From their first introduction in 2011, this approach, in which antibodies are used to block key inhibitory receptor checkpoints CTLA-4, PD-1 on T cells, and PD-L1 on tumour cells, has led them to be used as first-line treatments for several cancers, with more clinical trials in progress. Anti-PD-1 antibodies have been at the forefront of this success but the first of the ICI to be introduced, anti-CTLA-4 antibodies, have faced difficulties preventing broad therapeutic application, despite evidence of an enduring anti-tumour immunity in some cancers, notably melanoma. This review will examine how antibodies specific for CTLA-4 enhance anti-tumour immunity and highlight a largely unrecognized novel function, masked by current anti-CTLA-4 antibodies.

## The PD-1/PD-L1 antibodies—model checkpoint inhibitors

The anti-PD-1/PD-L1 antibodies are efficacious, clearly demonstrating that the strategy of simple antibody blockade of checkpoint receptor activity induces profound clinical benefits [3–12]. The premise of their activity came following initial analyses of tumour tissue biopsies showing increased tumour cell expression of the PD-1 ligands, PD-L1, and PD-L2 [13–17]. In turn, this revealed an immune escape mechanism, which could be blocked by targeting either the PD-1 receptor on T cells or PD-L1 on cancer cells. Identification of this immune evasion strategy from tumour tissue biopsy staining also revealed PD-L1 to be present in varying amounts, an observation which now supports its use as a biomarker to stratify patients likely to be responsive to anti-PD-1/PD-L1 therapies [18]. There remains, however, a need for improved patient stratification biomarkers and alternative options for patients unresponsive to PD-1/PD-L1 therapies.

Of the seven anti-PD-1 antibodies now approved for use by the FDA/EMA (Table 1), all of them use the IgG_4_ subclass backbone, to avoid T-cell toxicity through Fc-dependent effector functions. Thus, a simple blockade of function is the aim, and these antibodies achieve that. For the four approved anti-PD-L1 antibodies, it is slightly more complicated; atezolizumab was developed as an IgG_1_ antibody, but aglycosylated through an N297A mutation that limits antibody-dependent cell-mediated cytotoxicity (ADCC) or antibody-dependent cell-mediated phagocytosis (ADCP) [5]. Durvalumab, another IgG_1_ anti-PD-L1 antibody has three Fc domain mutations that also prevent ADCC/ADCP [7]. In contrast, avelumab is an unmodified IgG_1_ antibody, which has the potential to induce ADCC and ADCP [6]. In 2024, sugemalimab, the first anti-PD-L1 antibody to use the IgG_4_ format, was approved for the treatment of non-small-cell lung cancer (NSCLC) [19]

**Table 1.** Currently approved checkpoint inhibitor antibodies

Name	Antibody subclass	First approved(year)	ADCC/ADCP potential	Notes
CTLA-4				
Ipilimumab	IgG_1_	2011	**✓✓✓✓✓✓**	
Tremelimumab	IgG_2_	2022	**✓✘**	Approved for use in combination with durvalumab (unresectable hepatocellular carcinoma) or in combination with durvalumab and platinum-based chemotherapy (non-small-cell lung cancer)
PD-1				
Nivolumab	IgG_4_	2014	**✓✘**	
Pembrolizumab	IgG_4_	2014	**✓✘**	
Cemiplimab	IgG_4_	2018	**✓✘**	
Dostarlimab	IgG_4_	2021	**✓✘**	
Retifanlimab	IgG_4_	2023	**✓✘**	
Toripalimab	IgG_4_	2023	**✓✘**	
Tislelizumab	IgG_4_	2023	**✓✘**	
PD-L1				
Atezolizumab	IgG_1_	2016	**✓✘**	Fc disabled via an N297A mutation to remove glycosylation—allows binding only to FcgRI
Durvalumab	IgG_1_	2017	**✘**	Fc disabled via a triple mutation (L234F/L235E/P331S) to prevent all FcgR binding
Avelumab	IgG_1_	2017	**✓✓✓✓✓**	
Sugemalimab	IgG_4_	2024	**✓✘**	
LAG-3				
Relatlimab	IgG_4_	2022	**✓✘**	Approved for use in combination with nivolumab for malignant melanoma

The simple antibody blockade approach of these antibodies combined with a biomarker that supports clinical decision-making has rendered the PD-1/PD-L1 antibodies hugely successful in the treatment of many cancers. Currently, they are in more than 3000 clinical trials for a broad range of cancers, either as mono- or combination therapies. There are 5 more anti-PD-1/PD-L1 antibodies under regulatory review and at least 10 more in late-stage development [20]. Global sales of PD-1/PD-L1 checkpoint inhibitor therapies exceed $40 billion per annum despite their relatively recent first introduction in 2014. Simple antibody blockade works for the PD-1/PD-L1 axis and they therefore represent ‘model’ checkpoint inhibitors but this is not the case for anti-CTLA-4 antibodies, which although influencing the naïve T-cell response at a more fundamental level than the PD-1/PD-L1 axis have seen less clinical success.

## Characteristics of CTLA-4

CTLA-4 was first described as a receptor in activated cytotoxic T cells [21]. In humans, the CTLA-4 gene (*CD152*) is located on chromosome 2q33 [22] next to the partially homologous gene encoding the CD28 costimulatory receptor. During naïve T-cell activation, CD28 provides the costimulatory signal by binding its ligands CD80/CD86 on antigen-presenting cells to induce full activation of the cognate, antigen-specific T-cell response mediated by T-cell receptor recognition of peptide antigen presented by Class I or II MHC molecules [23]. CTLA-4 competes with CD28 to modulate or suppress T activity after activation by binding CD80/CD86 [24]. CTLA-4 binds both CD80 and CD86 with higher affinity and avidity than CD28 allowing dominion of the inhibitory response and therefore plays an important role in maintaining immune homeostasis and tolerance to self-tissues [24, 25] The importance of CTLA-4 was demonstrated by CTLA-4 knockout mice, which die postnatally from activated immune T-cell infiltrates in tissues and organs [26, 27]. A soluble fusion protein form of CTLA-4, CTLA4-Ig, rescues these CTLA-4^KO^ mice [28] and chimeric mice carrying a mix of effectively CTLA-4^+^ and CTLA-4^KO^ T cells are also rescued [29]. In adult mice rendered CTLA-4 deficient, autoimmune pathology is present but not lethal [30]. From these experiments, we interpret that CTLA-4 plays an active role in immune homeostasis in two distinct ways. CTLA-4 expressed on effector T cells intrinsically controls the activation status of the individual cell, but CTLA-4 also plays a broader role by controlling activated cell populations in an extrinsic manner.

## The role of CTLA-4 in regulatory T cells (T_REG_)

Control of activated effector T-cell populations by CTLA-4 is most likely mediated by T_REG_ which, unlike effector T cells, express CTLA-4 constitutively and at higher cell surface levels [31, 32]. The FoxP3 transcription factor, which is the defining signature marker of regulatory T cells, controls the potency of the regulatory function [33]. Whether naturally expressed or induced, Foxp3 mediates the suppressive function of T_REGs_ through multiple mechanisms including the secretion of inhibitory cytokines, the depletion of IL-2 through their high expression of its receptor CD25, the selective hydrolysis of the energenic molecules ATP/ADP or active production of immunosuppressive cytokines and constitutive surface expression of CTLA-4 [34]. The role of CTLA-4 in T_REG_ function is of relevance to cancer immunotherapy, where inhibiting T_REGs_ has been shown to improve anti-tumour immune responses and prognosis both in animal models and in clinical studies. Multiple strategies have been studied for targeting T_REGs_ such as depleting CD4^+^CD25^+^ cells using anti-CD25 antibodies [35], selectively inhibiting T_REGs_ through targeting the PI3K/Akt pathway [36, 37], low-dose cyclophosphamide, and as a direct result of ICI targeting PD-1/PDL-1 and CTLA-4 (alone or in combination with other therapies) [38]. Depleting T_REGs_ within the TME is, therefore, of importance, and targeting CTLA-4 antibody either to block CTLA-4 function or to actively target and deplete T_REGs_ represent valid strategies for inducing potent anti-tumour activity.

In terms of how CTLA-4 on T_REGs_ functions physically to inhibit activated effector T-cell populations and the immune response, there is evidence that CD80/CD86 removal from APC cell surfaces is mediated by transendocytosis in which CTLA-4 binds to and internalizes CD80/CD86 ligands to suppress indirectly the costimulatory signal [39, 40]. Removal of CD80 also exposes and increases PD-L1 levels to further inhibit T-cell responses by binding PD-1 [41]. Other recognized mechanisms mediated by CTLA-4 include induction of the kynurenine pathway and release of indoleamine 2,3 dioxygenase to reduce the availability of the essential amino acid tryptophan [42], or simply CTLA-4-mediated blockade of CD80/CD86 ligand availability to its costimulatory homologue, CD28 [43].

## Anti-CTLA-4 antibodies—the first checkpoint inhibitors

Antibodies to CTLA-4 have made a successful transition from bench to bedside for the treatment of several cancers, but there is uncertainty about how they achieve their anti-tumour effects. In fact, there was uncertainty regarding anti-CTLA-4 antibody function before the emergence of ipilimumab as the first ICI in 2011 [44]. In contrast with the notion of inducing anti-tumour activity, initial anti-CTLA-4 blockade data were ambiguous with evidence that anti-CTLA-4 mAbs enhanced mixed lymphocyte responses but also suppressed effector T cells activated by agonist anti-CD3 and CD28 mAbs [45, 46]. In another study, anti-CTLA-4 antibodies actually induced apoptosis of activated T cells [47]. Anti-CTLA-4 antibody Fab fragments, however, did not suppress T-cell activity, supporting the notion that a CTLA-4 crosslinking effect underpinned the suppressive mechanisms of anti-CTLA-4 antibody activity [46]. In effect, anti-CTLA-4 antibodies have some capacity to act as activational antagonists by enhancing the inhibitory effects of CTLA-4 function, contrasting with the costimulatory agonist effects mediated by most anti-CD28 antibodies [48].

Contrasting with these studies of anti-CTLA-4 antibody-mediated inhibitory effects on T-cell activation were clear demonstrations of anti-tumour activity in several mouse models, supporting the potential for treating patients with anti-CTLA-4 antibodies [49–53]. One notable observation was that in addition to anti-tumour activity, the application of anti-CTLA-4 antibodies established a specific protective memory response against the tumour following treatment with anti-CTLA-4 antibodies, opening the possibility of a post-tumour vaccination effect [49, 50, 53]. Critical to the productive anti-tumour immune response were T cells, and in the B16-BL6 melanoma model, CD8^+^ T cells [53]. This uncertainty of how anti-CTLA-4 antibodies on one hand appear to actively inhibit T-cell responses, but nevertheless have the capacity to augment T-cell-mediated anti-tumour responses is difficult to reconcile, and on top of that there is less information regarding effects on human T-cell-mediated immunity *in vitro.* One study, however, has shown that anti-CTLA-4 antibodies do also inhibit antigen-specific T-cell responses [54].

## Ipilimumab, the first human anti-CTLA-4 checkpoint inhibitor

Ipilimumab, a fully human IgG_1_ anti-CTLA-4 antibody, was first approved for use in 2011, initially as a novel therapy for Stage 4 malignant melanoma [44]. Ipilimumab was the first therapy to show a clear increase in both overall survival and progression-free survival in advanced melanoma patients for over 30 years [55]. It heralded a new dawn for the treatment of melanoma, and in parallel with the anti-PD-1/PD-L1 therapies, for cancers more generally. There was, however, a curious caveat. Patients receiving anti-CTLA-4 therapy sometimes exhibit a delayed response for some weeks or months before anti-tumour immunity becomes apparent [56]. In the absence of a response biomarker, this manifestation contributes to clinical uncertainty, especially as it can take the form of pseudo-disease progression as well as a simultaneous mix of disease progression and response. Why does it take so long for the adaptive immune system to mobilize anti-tumour efficacy? This is not the case for the anti-PD-1/PD-L1 antibodies where late responders are less frequently observed, at least in melanoma [57]. Despite this clinical uncertainty, analysis of malignant melanoma patients responsive to anti-CTLA-4 ipilimumab therapy who were monitored over a period of more than 10 years revealed that they appear to have gained long-term protection, supporting the possibility of a post-tumour vaccine effect [58].

Currently, the only other anti-CTLA-4 antibody approved for clinical use is tremelimumab, a human IgG_2_ antibody, that received orphan drug designation for the treatment of mesothelioma in 2015, and more recently was approved as a combination therapy with durvalumab (anti-PD-L1) for adult patients with unresectable hepatocellular carcinoma [59] and further, in combination with durvalumab and platinum-based chemotherapy for a subtype of metastatic NSCLC [60]. Despite its late approval compared with ipilimumab, tremelimumab appears to be better tolerated, as a lower frequency of hypophysitis was observed in a clinical trial involving melanoma patients than typically seen with ipilimumab, likely due to reduced complement activation by the IgG_2_ antibody subclass [61]

## The CTLA-4 antibodies—releasing the brakes or eliminating T_REGs_ in murine models

**Figure 1.**
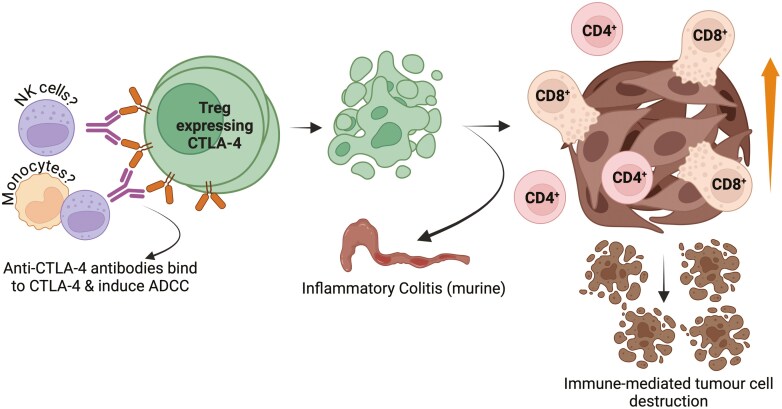
Anti-CTLA-4 antibody-dependent T_REG_ apoptosis increases immune-cell-mediated tumour destruction in murine models. Antibodies specific for CTLA-4 bind the CTLA-4 receptor, which is expressed at high levels on T_REG_ within the tumour microenvironment and induce ADCC to render them apoptotic. This results in an increase in immune effector cell activity, leading to tumour destruction. The requirement for ADCC mediated T_REG_ destruction is Fc domain dependent, which is also associated with bystander autoimmunity, especially gut inflammatory responses (https://biorender.com/i67o286).

How do anti-CTLA-4 antibodies achieve their function? It is not straightforward. Originally, a simple blockade of CTLA-4 was proposed, with the notion of ‘releasing the brakes’ by boosting the anti-tumour T-cell response [62]. Several groups, however, have used murine models to evaluate the mechanism of anti-CTLA-4 activity, providing evidence that the killing of immune cells is required for anti-tumour activity to occur (Figure 1) [63–72]. These target cells are posited to be intratumoural T_REG_, thus, anti-CTLA-4 antibodies bind CTLA-4, expressed at higher levels on this T_REG_ subset, consequently eliminating them via Fc-receptor-mediated ADCC, and allowing an effector T-cell response to dominate. The antibody Fc domain is, therefore, critical to their anti-tumour function, because reformatting antibodies onto a benign subclass or Fc silent background, completely abolishes their efficacy [63–66, 68–71]. Most murine anti-CTLA-4 antibodies utilize the more aggressive IgG_2a_ or IgG_2b_ Fc backgrounds that when switched to a benign IgG_1_, Fc silent, or are- removed altogether, limis any capacity for Fc-receptor-mediated ADCC. This contrasts with the approach taken with anti-PD-1 antibodies, which do not rely on an Fc domain that can mediate ADCC (Table 1). Which type of immune cells induce this intratumoural T_REG_ obliteration is not well-defined, but NK cells and atypical monocyte/macrophage subsets have been identified as the most likely contenders, based on intratumoural availability and relatively high expression of effector Fc receptors [65–67, 72]. A corollary to these murine studies of anti-CTLA-4 Fc effector dependency is the effects on gastrointestinal inflammation, which in concert with a loss of anti-CTLA-4 efficacy, are also reduced when an ADCC-inducing antibody Fc subclass is replaced with a benign or silent Fc background [73].

**Figure 2.**
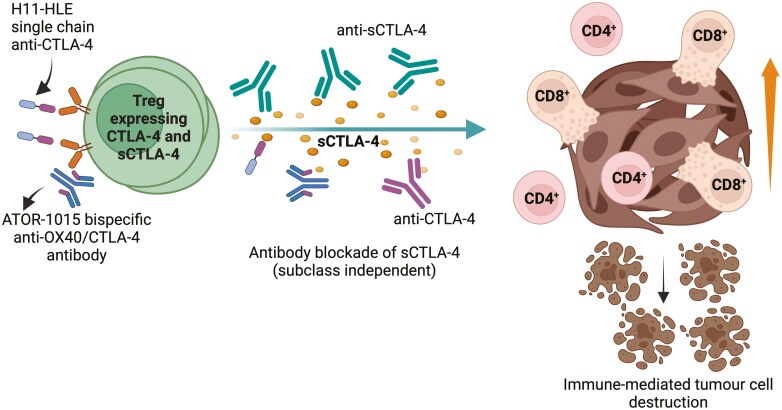
Inhibition of CTLA-4 or sCTLA-4 induces anti-tumour immunity. Non-depleting antibodies specific for CTLA-4 or antibodies selective for the soluble sCTLA-4 isoform, can induce tumour destruction without destruction of T_REG_ (https://biorender.com/r45t001).

One further study, however, demonstrated clear anti-CTLA-4-mediated anti-tumour activity that did not require FcgR-mediated destruction of T_REG_. Sato *et al*. used H11-HLE, a high-affinity-variable heavy-chain fragment, derived from a heavy-chain-only alpaca anti-CTLA-4 antibody. In both MC38 (colon adenocarcinoma) and H22 (hepatoma) mouse tumour models they revealed anti-tumour activity, which was Fc independent [74]. Further, they also indicated that the human antibody, ipilimumab, required Fc effector function for efficacy in the murine MC38 model, but observed a retained anti-tumour activity in the H22 model when its Fc effector function was ablated [74]. Similarly, a human anti-OX40 antibody, ATOR-1015, rendered bispecific by attachment of anti-CTLA-4 Fab domains, modified from CD86, to the C terminal anterior of each light chain, demonstrated both the ability to enhance antigen-specific immune responses and to deplete T_REG_ in human OX40 ‘knock in’ hOX40tg mice [75]. Overall, these latter studies hint at two separate mechanisms of anti-CTLA-4-mediated anti-tumour immunity, the first dependent on T_REG_ depletion (Figure 1), but the second more akin to the concept of ‘releasing the brakes’ in which simple blockade is capable of generating productive immunity (Figure 2).

## The CTLA-4 antibodies—releasing the brakes or eliminating T_REGs_—human studies

In humans, it is less clear whether T_REG_ ablation is required for efficacy. It is, of course, difficult to study such aspects in patients, but there is some evidence for both T_REG_ elimination and activation via simple antibody-mediated CTLA-4 blockade. An initial study in which human nonclassical monocytes (CD14^+^CD16^++^) from patients responsive to ipilimumab were examined, highlighted an increase in peripheral numbers of this monocyte subset compared with unresponsive patients, together with a demonstration of direct T_REG_ killing in the presence of ipilimumab, *in vitro* [76]. Analysis of the TME from matched biopsies of patients treated with ipilimumab, however, indicated that T_REG_ depletion was not necessary for efficacy [77]. Analysis of a mouse model, in which human anti-CTLA-4 antibodies were investigated using humanized Fc receptors, revealed that both ipilimumab and tremelimumab have the potential to eliminate T_REG_ from the TME via ADCC [67]. Further analysis with Fc-region-modified anti-CTLA-4 mAbs to enhance ADCC capacity also provided support for T_REG_ depletion as an important mechanism of action, but further, indicated a concurrent reduction in effector CD8^+^ T-cell numbers [78, 79] suggesting that activated effector cells expressing CTLA-4 might also be deleted.

## The soluble isoform of CTLA-4

Most CTLA-4 research and clinical translation have focused on the full-length CTLA-4 receptor isoform, but other isoforms exist and contribute to immune regulation [80–83]. In mice, there are four isoforms comprising the full-length CTLA-4 encoded by exons 1, 2, 3, and 4, ligand-independent CTLA-4 (liCTLA-4; exons 1, 3, and 4), a secretable soluble isoform (sCTLA-4; exons 1, 2, and 4) and small peptide fragment encoded by exons 1 and 4 [81]. In humans, liCTLA-4 is not encoded [83]. The C terminal domain of sCTLA-4 and the peptide fragment is affected by a frameshift during translation giving rise to a different amino acid sequence compared to full-length CTLA-4. Of note, a cysteine residue at position 120, responsible for homodimerization of the CTLA-4 receptor [84], is lost in sCTLA-4, although another is gained at position 128 [81].

Initially, sCTLA-4 was considered to be produced as a monomeric isoform and was assumed to have reduced regulatory activity compared with its receptor counterpart. Recent data, however, provided good evidence that sCTLA-4 is secreted in homodimeric form [85], and further, can bind CD80 via an RGMB receptor, to enhance its inhibitory function [86]. Other studies also confirm the functional relevance of sCTLA-4 produced by activated T_REG_ in suppressing T-cell responses [87], but further, in altering the phenotype of any active immune milieu toward a Th2/M2 reparative environment, which is immune suppressive and cancer promoting [88].

The influence of sCTLA-4 in serum and the TME is important because current anti-CTLA-4 antibodies can bind it and therefore high levels of sCTLA-4 can effectively impinge upon optimum dosing, a factor that is largely ignored in current therapeutic regimes. High serum levels of both sCTLA-4 and sPD-L1 identified patients with NSCLC r or gastrointestinal cancer less likely to respond to anti-PD-1 therapy, emphasizing the importance of this largely overlooked alternatively spliced isoform of CTLA-4 [89, 90]. Prior to that, a melanoma study revealed a disparity in ipilimumab response success correlating with sCTLA-4 serum levels, with high serum levels above 200 pg/mL pointing to a better patient outcome [91]. In our own human studies, we identified sCTLA-4 to be raised in a melanoma patient cohort and was immunosuppressive, comparable to artificial CTLA4-Ig [87]. In a large study of lupus patients, we found that selective blockade of sCTLA-4 also enhanced cytokine secretion following activation [92]. Further, a study of sCTLA-4 as a potential biomarker demonstrated expression patterns in oral and oropharyngeal epithelial tissues with the potential to identify premalignant dysplasia preceding development of head and neck neoplasia [93]. Finally, we find that selective blockade of sCTLA-4 enhances antigen-specific immune responses but has little effect on resting cells [94].

But could sCTLA-4 play a direct role in tumour immunity either as a novel immune evasion mechanism or as a viable target for therapy? This alternatively spliced isoform is indeed produced in some cancers. Primary melanoma cell lines were identified to produce sCTLA-4 [95] and further, mesothelioma cells were also closely associated with its production [96]. It has also been reported present by transcriptome analysis and confocal microscopy in several other cancers and cancer cell lines [86, 87].

Because the C terminal sequence of sCTLA-4 differs from that of full-length CTLA-4, we generated selective anti-sCTLA-4 antibodies [94]. In previous work, we demonstrated anti-tumour activity in the murine B16-F10 metastatic melanoma model and have identified activity in other mouse models [85, 94]. The anti-tumour activity is consistent and predictable, and moreover, by their very nature, these murine anti-sCTLA-4 antibodies cannot induce ADCC because they do not target cells and have a benign murine IgG_1_ Fc backbone, approximately equivalent to human IgG_4_. Therefore, this is unlike the majority of anti-CTLA-4 antibodies that do require an ADCC inductive antibody subclass, and whose activity is lost upon replacement with a benign Fc background such as murine IgG_1_ [63–72]. This raises the prospect of explaining anti-tumour activity, which is not dependent on T_REG_ depletion and is in line with the concept of ‘releasing the brakes’ (Figure 2).

How then do we explain these discrepancies in activity between anti-CTLA-4 and anti-sCTLA-4 antibodies? Perhaps the most controversial element to address is that of antigen specificity. There is very little evidence indicating that CTLA-4 blockade with anti-CTLA-4 antibodies can induce potent antigen-specific immune responses *in vitro*. In fact, quite the opposite, with good evidence that crosslinking CTLA-4 on activated T cells is actively suppressive [46, 54, 94]. Selective blockade of sCTLA-4 consistently enhances antigen-specific responses in terms of cell proliferation and cytokine production *in vitro*, which would surely be beneficial in a clinical setting, especially in boosting tumour-associated vaccines [96–99]. Regarding anti-tumour activity in murine models, both anti-CTLA-4 and selective anti-sCTLA-4 antibodies can induce efficacious immunity (see above). Both types of antibodies are functional in some models, for example, the B16-F10 metastatic melanoma model, but do not overlap precisely. We propose that while the direct killing of T_REG,_ mediated by anti-CTLA-4 antibodies, is the primary mechanism through which they exert their anti-tumour effects, selective blockade of sCTLA-4 contributes to ‘releasing the brakes’ of the antigen-specific immune response (Figures 1 and 2). Thus, there are two, and to some extent, mutually exclusive routes to anti-tumour immunity. Because anti-CTLA-4 antibodies bind both CTLA-4 receptor and sCTLA-4, patient responses may also vary, depending upon levels of sCTLA-4. For instance, if most anti-CTLA-4 antibodies are binding sCTLA-4, the blockade route to productive immunity would be favoured, whereas if the CTLA-4 receptor was predominantly bound that would favour the T_REG_ depletion route. Two recent retrospective studies of anti-PD-1 activity in advanced NSCLC and gastric cancer patients found that combinations of soluble serum factors including high levels of sCTLA-4 were predictive of a poor response to therapy [90, 91]. It seems likely, given the immunosuppressive nature of sCTLA-4, this would also apply to other checkpoint inhibitor therapies.

In summary, there is good evidence that anti-CTLA-4 mabs have two separate routes to achieving productive cancer immunotherapy, either via T_REG_ depletion (Figure 1) or simple blockade of sCTLA-4 or the CTLA-4 receptor (Figure 2). It is therefore crucial to understand and reconcile the conflict between these two mechanisms to further enhance our understanding of immune homeostasis and tolerance, as well as further development of safer, efficacious ICI therapies.

## Data Availability

Not applicable. This is a review article covering already published primary data.

## Permission to reproduce

Both Figure 1 and Figure 2 were created with BioRender. Figure 1 can be found at: https://biorender.com/i67o286 and Figure 2 can be found at: https://biorender.com/r45t001
